# Miss Shannon and the Grantham Infirmary

**Published:** 1895-02-16

**Authors:** 


### MISS SHANNON AND THE GRANTHAM HOSPITAL.

We have received the following communication from an authoritative source, which we publish with great pleasure: "I send you per post the annual report of the Grantham and District Hospital. We, like most other hospitals, have had bad times; but two years ago we were fortunate enough to secure the services of Miss Shannon as matron, and it is mainly through her ability and thoroughness that we now flatter ourselves we are one of the best-managed country hospitals. Since Miss Shannon has been here, the hospital has regained its popularity, the subscriptions have increased, the presents have increased, the number of patients have increased, the expenditure has decreased, and she continues to gain golden opinions from all classes. The Committee of Management express in their report a wish to again place on record their appreciation of the manner in which Miss Shannon has discharged her duties; and at the annual general meeting, held on Thursday last, the Earl of Brownlow, lord-lieutenant of the county, in the chair, Canon Welby, one of the senior governors and a member of the House Committee, moved a special vote of thanks to Miss Shannon, saying, 'how very satisfactory the work of the year had come out, due, he knew, to the exertions of their excellent matron.' They had had considerably more patients in the hospital, and yet the cost had been considerably less; the care and economy she had shown in the working of the hospital deserved special praise, and the kindness and courtesy to all with whom she came in contact led him to propose that a vote of thanks to Miss Shannon be entered upon the minutes. The Rev. Father Sabela, in seconding the resolution, said he could endorse every word uttered by Canon Welby with regard to the matron; she was very painstaking, and had done everything in her power to make the patients comfortable. He thought, therefore, she deserved all the praise bestowed upon her by Canon Welby. I send you this on the principle of giving honour to whom honour is due, and think that a little more public recognition of the services of really good matrons and nurses would stimulate emulation in others."

